# How Archer Fish Achieve a Powerful Impact: Hydrodynamic Instability of a Pulsed Jet in *Toxotes jaculatrix*

**DOI:** 10.1371/journal.pone.0047867

**Published:** 2012-10-24

**Authors:** Alberto Vailati, Luca Zinnato, Roberto Cerbino

**Affiliations:** 1 Dipartimento di Fisica, Università degli Studi di Milano, Milano, Italy; 2 Dipartimento di Biotecnologie Mediche e Medicina Traslazionale, Università degli Studi di Milano, Segrate (MI), Italy; University of Ottawa, Canada

## Abstract

Archer fish knock down insects anchored to vegetation by hitting them with a precisely aimed jet of water. The striking force of the jet at the impact is such to overcome the strong anchoring forces of insects. The origin of the effectiveness of such hunting mechanism has been long searched for inside of the fish, in the unsuccessful attempt to identify internal structures dedicated to the amplification of muscular power. Here we perform a kinematic analysis of the jet emitted by two specimens of *Toxotes jaculatrix*. We estimate that at the impact the jet conveys a typical specific power of about 3000 W/kg, which is well above the maximum specific power of the order of 500 W/kg deliverable by a vertebrate muscle. Unexpectedly, we find that the amplification of muscular power occurs outside of the fish, and is due to a hydrodynamic instability of the jet akin to those occurring in Drop-on-Demand inkjet printing. The investigated fish are found to modulate the velocity of the jet at the orifice to favor the formation of a single, large, water drop that hits the prey abruptly with a large momentum. The observed mechanism represents a remarkable example of use of an external hydrodynamic lever that does possibly not entail the high evolutionary cost needed for the development of highly specialized internal structures dedicated to the storing of mechanical energy.

## Introduction

Archer fish have developed a unique method for capturing the insects populating the canopy of vegetation overhanging the mangrove swamps where they live. Once that they spot their prey, they shot it down by squirting a precisely aimed jet of water with their mouth, so that the prey falls into water where it gets readily devoured. Preys are usually firmly anchored to vegetation, with anchoring forces being typically of the order of ten times their body weight [1]–[3]. The origin of the effectiveness of the predation mechanism in archer fish has been long debated [4]–[11], since the first account on the hunting behaviour of archer fish in 1764. The striking force of the jet at the impact suggested that the squirting could be driven by internal structures able to amplify muscular power [9], so to overcome the strong anchoring forces of insects. Examples of structures dedicated to the amplification of muscular power are present in chameleons [12]–[14] and salamanders [15], [16], where the muscular energy gets slowly stored into collagen fibers, and then released abruptly to project their tongues with acceleration as high as 500 m/s^2^. Similar structures have been searched for inside of archer fish [9], but accurate morphological analysis and electromiography measurements on the muscles involved in the squirting process ruled out their presence [10]. The problem of the origin of the effectiveness of the jet remains thus still unsolved.

By using a combination of high-speed video recording and kinematic analysis of the acquired video sequences, we performed a systematic experimental investigation of the water jet squirted by two specimens of *Toxotes jaculatrix* (Pallas, 1767). We found that, during the propagation of the jet to the prey, the velocity of the jet at the mouth of the fish is modulated so to achieve a gradual increase of the mass accumulated at the head of the jet and of its velocity. As a result, the power that the head of the jet can deliver upon impact increases during the shooting. When the jet hits the prey the mass-specific power carried by the jet has grown to a typical value of about 3000 W/kg, irrespective of the distance of the prey and of the shooting angle. This figure largely exceeds the maximum specific power of about 500 W/kg deliverable by a vertebrate muscle [17], [18], suggesting the importance of the observed external amplification mechanism in the effectiveness of the hunting strategy adopted by archer fish.

## Results

**Figure 1:**
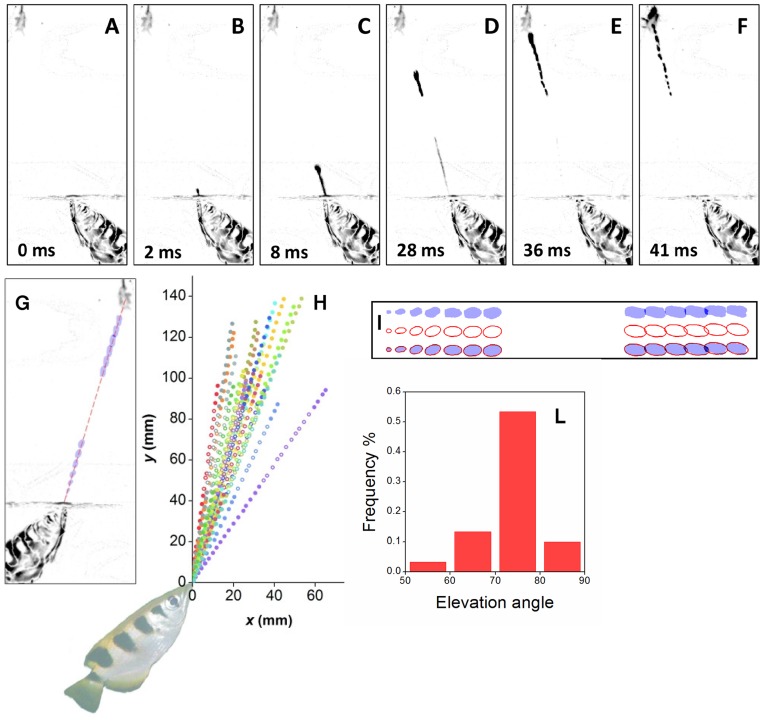
Propagation of the jet. (A–C) acceleration phase. (D–E) nearly ballistic phase. (F) impact. (G) progressive increase of the size of the head of the jet during the flight to the prey. The dashed line outlines the linear trajectory of the head of the jet. The horizontal band free of data at the mid-height of the figures delimits the region where the observation of the jet was prevented by the presence of a slit needed to achieve a lateral view of the jet [19]. (H) trajectories of the head of the jet. Open points correspond to interpolated data in the region where the observation of the jet was prevented by the slit. All the data are compatible with a linear trajectory, with correlation coefficients in the range 0.99622<r^2^<0.99994. (I) interpolation of the head of the jet with ellipsoids. Top: projection of the head of the jet. Middle: interpolating prolate ellipsoids. Bottom: superposition of the projection and the ellipsoids. (J) distribution of shooting angles.

**Figure 2:**
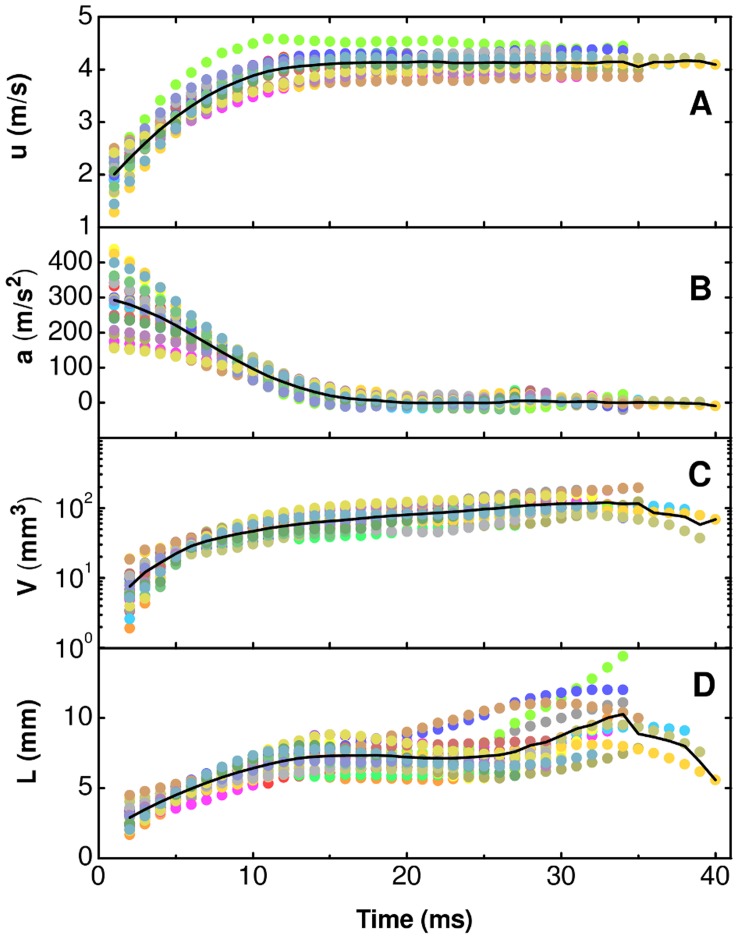
Kinematics of the head of the jet. (A) Time evolution of the velocity, (B) of the acceleration, (C) of the volume, and (D) of the axial length of the head of the jet. Data refer to the trajectories reported in Fig. 1H and the same color coding is adopted here. Continuous lines represent averages on all sequences.

Archer fish are able to hit aerial preys with a powerful jet of water in a fraction of a second. The investigation of the mechanism leading to the effectiveness of the impact requires the utilization of a technique able to characterize the force and power delivered by the jet. In principle, the use of a strain gauge would allow the direct measurement of the force at the impact. However, such a solution would not allow to investigate the dynamics of the jet during its propagation to the prey, which proves to be essential to understand the physical mechanism that leads to a powerful impact. Therefore, in order to attain a time resolved characterization of the jet we employed a high-speed video recording technique. This non-invasive diagnostics allows us to determine the kinematics of the jet and to estimate the time evolution of the characteristic size and volume of different parts of the jet. The main requirement of the method is the availability of lateral movies of the jet, free from distortions due to the refraction at the lateral walls of the tank hosting the fish. Lateral movies are obtained by obliging fish to align parallel to a lateral window of the tank when they squirt a jet of water to prey. The alignment of fish has been obtained by placing a narrow slit on top of the water tank, so that they are able to localize the prey precisely with both eyes only when their sagittal plane is parallel to the side of the tank used as observation window [19]. A typical lateral-view image sequence of the flight (Video S1) of the jet to the prey involves an initial acceleration phase (Fig. 1A–C), followed by a nearly ballistic phase (Fig. 1D–E), and by the impact (Fig. 1F). The jet appears as being composed of a thin tail and a bulged head, with the volume of the head of the jet progressively increasing during the flight (Fig. 1G, I). In the investigated range of shooting distances (97–153 mm), the motion of the head of the jet is compatible with a linear trajectory (Fig. 1H), independently of the shooting angle. The distribution of the elevation angle of the jet above the horizon (Fig. 1J) peaks around 74°. Data for the velocity and acceleration of the head of the jet as a function of time (Fig. 2A–B) have been obtained from the same shooting sequences of Fig.1H. The jet of water is ejected from the mouth of the fish with a typical velocity of about 2 m/s. At such small velocities the drag of the surrounding air on the jet can be neglected [20]. Initially the head of the jet undergoes a strong acceleration phase, where the acceleration drops from 200–400 m/s^2^ to zero in about 15 ms. This phase is followed by a nearly-ballistic phase lasting 20–30 ms. The initial acceleration brings the head to a velocity of about 4 m/s. The accelerated motion of the head of the jet and the increase of its volume indicate that during the acceleration phase the velocity of the tail of the jet is larger than that of the head. The head gets progressively inflated by the liquid incoming from the tail, which also provides the thrust responsible of the acceleration of the head. This behaviour of the jet is achieved by modulating the velocity at the orifice, so that it increases gradually at the beginning of the emission of the jet. Incidentally, the modulation of the velocity at the orifice does not require specialized structures and is intrinsic in the dynamics of the spitting process: quite generally, during the pulsed emission of liquid from a nozzle the liquid starts at rest and its velocity increases gradually during the leading part of the pulse. The fact that the initial velocity of the jet is not zero suggests that before spitting the fish inhales a small amount of air into the orifice. During the acceleration phase the trajectory is not significantly affected by gravity. At the impact, vertical deviations from a linear trajectory due to the presence of gravity are estimated to be of the order of 0.5 mm, corresponding to about 10% of the size of the head of the jet. The fact that the trajectory of the head of the jet is linear allows a fast optimization of the aiming process.

**Figure 3:**
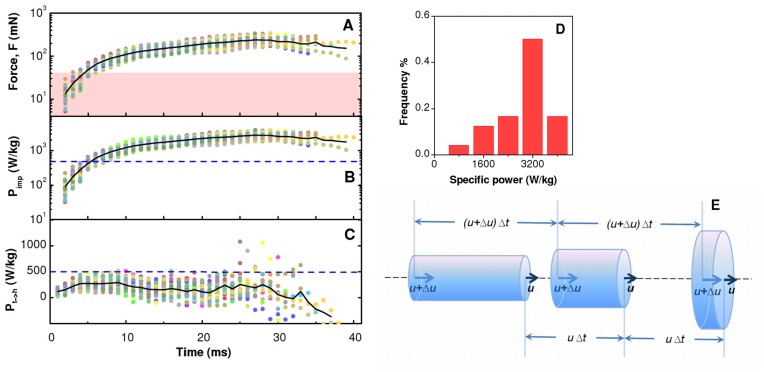
Force and power at the head of the jet. (A) time evolution of the maximum average force that the jet head can exert at the impact. The shaded area indicates the range of typical anchoring forces of insects such as flies, bugs and beetles [1]–[3]. (B) time evolution of the mass-specific power that would be required by the muscles involved in the emission of the jet to accelerate the head in the absence of the hydrodynamic amplification process discussed here. (C) time evolution of the mass-specific power transfereed instantaneously to the head of the jet. The horizontal dashed line in panels (B) and (C) represents the limit of 500 W/kg for a vertebrate muscle [17], [18]. Data refer to the trajectories reported in Fig. 1H and the same color coding is adopted here. Solid lines in (A), (B) and (C) represent averages on all sequences. (D) Distribution of the mass-specific power at the impact. The median of the distribution is 2950 W/kg. (E) The motion of a cylindrical section of the jet is shown for three time instants separated by Δ*t*. The acceleration of the liquid at the orifice determines a propagation velocity *u* of the leading face smaller than the velocity *u* +Δ*u* of the trailing face. The velocity difference Δ*u* determines a shrinking of the length of the section and, in turn, the divergence of its radius.

The impact can be modelled as the collision of a droplet with a dry, solid target, in the absence of rebound, a problem in hydrodynamics that is still understood only partially [21], [22]. The impact of a droplet onto a dry, solid surface depends from many parameters, such as the droplet size and velocity, the surface tension and rheological properties of the liquid, the orientation, hardness, roughness, wetting properties and chemical composition of the surface of the target [21], [22]. In the case of the impact of the jet squirted by archer fish, relevant experimental parameters, such as the angle of impact and the nature of the surface of the target, cannot be controlled *a priori*. Nevertheless, from the kinematic parameters related to the propagation of the head of the jet it is possible to estimate the maximum average force and power available at the impact. To this purpose, we assumed that the collision occurs under ideal conditions: the impact is normal to the surface of the target, the target is still during the collision, the surface of the target is completely wettable, and no rebound occurs during the collision. This gives rise to a completely inelastic collision, characterized by a momentum transfer Δ*p* = ρ*Vu* and by a maximum energy transfer Δ*K* = 0.5 ρ*V u* ^2^. To determine the force at the impact *F* =  Δ*p*/Δ*t_i_* we needed to estimate the impact time Δ*t_i_*. We assumed that Δ*t_i_* corresponds to the typical impact time [23], [24] Δ*t* = *L*/*u* needed by the head of the jet to travel a distance *L* corresponding to its axial length. Typical values of Δ*t_i_* are in the range 1–3 ms. During the propagation of the jet, the force *F* increases progressively (Fig. 3A, Fig. S1), due to the accumulation of mass at the head of the jet and to the increase of its velocity. The force at the impact has an average value of about 200 mN. The anchoring force of insects such as flies [2], and bugs [3] is typically smaller than 20 mN for specimens with a body mass up to 100 mg, with peak values of about 40 mN for beetles [1] (Fig. 3A, Fig. S1). Therefore, the force at the impact exceeds at least by a factor 5 the anchoring force of most preys. This ample margin guarantees the effectiveness of the collision even under non-ideal conditions encountered in practice. Our estimates of the force at the impact and of the adhesion force of insects represent upper bounds achievable under ideal conditions. During a real collision, both quantities are affected by a multitude of parameters, which can contribute to limit their values with respect to the upper bounds.

The power carried by the head of the jet can be calculated as *P* = Δ*K*/Δ*t_i._* The mass of the muscles involved in the emission of the jet can be estimated to be *m*_m_ *≈*178 mg for specimen 1, and 135 mg for specimen 2, and can be used to assess the mass-specific power *P*_imp_ = *P*/*m*_m_ that would be required to accelerate the head of the jet in the absence of the amplification mechanism (Fig. 3B, Fig. S1). It is interesting to compare the experimentally determined *P*_imp_ with the maximum mass-specific power that can be delivered by a vertebrate muscle, which is of the order of 500 W/kg [17]–[18]. At the emission of the jet the specific power carried by the head is fully compatible with direct muscular action. However, *P*_imp_ increases with time and saturates to a typical value of about 2950 W/kg for specimen 1 (Fig. 3D) and 2820 W/kg for specimen 2 (Fig. S1), thereby confirming the existence of an amplification mechanism with efficiency comparable with the catapult action in chameleons. The catapult mechanism in chameleons takes place internally to the animal, whereas the existence of an internal amplification mechanism in archer fish has been ruled out by previous studies [10]. The absence of an internal mechanism is also confirmed by our results, in that the instantaneous mass-specific power increase of the head of the jet remains well below 500 W/kg (Fig. 3C), with the exception of a few noisy points at large times. This result is compatible with the interpretation that power gets progressively transferred from the muscles to the tail of the jet, and in turn to the head of the jet, with no need of invoking amplification mechanisms internal to the fish. On the contrary, the striking finding is that such a slow accumulation of energy at the head takes place externally to the fish and leads eventually to a power at the impact that largely exceeds the one deliverable by the muscles involved in the emission of the jet.

## Discussion

Our results point to the existence of an external, hydrodynamic amplification mechanism. The understanding of the mechanism responsible of the amplification of power at the impact can be achieved in the framework of the physics of liquid jets.

### Dimensionless Numbers for Describing the Jet Stability

A cylindrical jet is intrinsically unstable with respect to perturbations at its surface. Some simple, yet effective, models have been developed for the destabilization of stationary jets. However, notwithstanding seminal work dating back to 150 years ago, the dynamics of a liquid jet is not yet completely understood [25], especially in the case considered here of the nonlinear behavior of pulsed jets, whose understanding still relies largely on empirical experimental and simulation work [26]. The stability of jets depends on a subtle balance of viscous forces, inertia and surface tension. Under our experimental conditions the Ohnesorge number, which relates the viscous forces to inertial and surface tension forces, is small: Oh = ν[ρ/(γ*r*_0_)]^1/2^≈3.7×10^−3^. The effects of viscosity can be thus neglected. The Weber number, which measures the relative importance of inertia compared to surface tension, is fairly large: We  =  ρ *r*_0_ *u*_0_^2^/γ≈120. Therefore, as a first approximation, the two most prominent contributions to the stability of the jet come from inertial effects and from surface tension, the first effect dominating onto the second.

### Rayleigh-Plateau Instability of a Stationary Jet

The contribution of surface tension to the destabilization of the jet can be best understood by considering the dynamics of a stationary cylindrical jet. The action of surface tension favors a configuration where the free surface of the jet is as small as possible. An axial modulation of the radius of the jet determines a reduction of the outer surface of the jet, provided that the wavelength of the modulation is large enough. Under these conditions for the stationary flow of an inviscid liquid the dominant mode in the destabilization of the jet (i.e. the unstable mode with the largest growth rate) corresponds to an axial modulation at the Rayleigh wave length λ_R_  = 9 *r*_0_, where *r*_0_ is the typical radius of the jet [27]. In the absence of external perturbations, the destabilization of the jet due to a Rayleigh-Plateau instability [20], [25], [27] would occur at the Rayleigh wavelength, and would break the jet into a stream of droplets of volume *V*_R_ =  π *r*_0_^2^ λ_R_ in a typical time τ_R_≈3(ρ *r*_0_^3^/γ)^1/2^. Here ρ is the mass density of the liquid, and γ its surface tension.

### Kinematic Gathering of a Pulsed Jet

For non-stationary flows, other modes can contribute to the destabilization of the jet. These modes can be selected by modulating the velocity of the jet at the orifice, as it happens during the emission of the jet in archer fish. An acceleration of the jet at the orifice determines an overall axial compression of the jet that causes the accumulation of mass and momentum at its head (Fig. 2A,C) through a kinematic gathering process [25], [28]. This process is similar to that occurring during the generation of droplets in Drop-on-Demand inkjet printing [26], [29], a technological field still largely driven by empirical developments that can potentially benefit from the biomimicry of archer fish. The amplification mechanism determined by kinematic gathering can be understood by considering the kinematic motion of a thin cylindrical section of the jet, of radius *r* and length *l* (Fig. 3E), and by neglecting the effect of surface tension and of body forces [25], [28]. We assume that the velocity at the orifice has a pulsed modulation *u*(*z* = 0, *τ*) =  *u*_0_[1+ ε *f*(*τ*)], where ε is a dimensionless number of the order of one and *f*(*τ*) is a pulse-shaped function consisting of a fast monotonic growth followed by a symmetrical decrease. During the rapid initial acceleration phase, the velocity *u* of the leading face of the cylindrical section becomes smaller than the velocity *u* + Δ*u* of the trailing face. Therefore, during the travel of the jet the two faces approach each other with a relative velocity Δ*u* = (∂*u*/∂*t*)|_τ_ Δ*t* and the length of the section shrinks in time as *l*(*t*)  =  *l*_0_ − Δ*u* (*t−τ*), where *l*_0_ = *u*(0, τ) Δ*t* is the length of the section at the time *τ* when it was emitted at the orifice (Fig. 3E). During the gathering process, the mass and momentum of the cylindrical sections are conserved, but their axial length becomes vanishingly small. As a result, the gathering of the sections gives rise to an overall axial compression of the jet during the acceleration phase. This axial compression of the jet determines a transfer of mass and momentum from the tail of the jet to its head. Conservation of volume of the cylindrical section implies that *r* ^2^ *l* = *r*_0_^2^ *l*_0_ and the radius evolves in time according to *r*(*t*)/*r*_0_ =  (*l*_0_/*l*)^1/2^ =  [1−Δ*u* (*t−τ*)*)/l*_0_]^−1/2^ =  [1−(∂*u*/∂*t*)|_τ_ (*t−τ*)*/u*(0, τ)]^−1/2^. At the time t^*^−*τ*  =  *l*_0_/Δ*u = u*(0, *τ*)/(∂*u*/∂*t*)|_τ_ the length *l* shrinks to zero and the radius of the cylindrical section diverges, in principle giving rise to the formation of a thin sheet of liquid perpendicular to the axis of the jet. The radial profile of the jet *r*(*z*)/*r*_0_ =  [1–*z*/*z* ^*^]^−1/2^ can be obtained by taking into account that at time *t* the section emitted at time *τ* has reached a distance *z* = *u*(0, *τ*) (t−*τ*) from the orifice. Here *z* ^*^ = *u* ^2^(0, τ)/(∂*u*/∂*t*)|_τ_ is the distance from the orifice where the radius of the jet diverges.

### Application to the Jet Squirted by Archer Fish

During the propagation of the jet in archer fish, both inertial effects and surface tension contribute to the destabilization of the jet. The relative influence of the two mechanisms on the destabilization of the jet can be established by comparing their typical time scales and length scales. For our experiments ρ = 1.01 g/cm^3^ is the mass density of brackish water, γ = 72.1 mN/m the surface tension and *r*_0_≈1 mm the typical radius of the jet at the orifice. With these values we obtain a Rayleigh wavelength λ_R_  = 9 *r*_0_≈9 mm, a jet breaking time τ_R_≈11 ms and a droplet volume *V*_R_ ≈28 mm^3^. The parameters related to the kinematic gathering can be estimated by considering that when the jet starts to be emitted by archer fish the velocity of the jet is *u*(0, 0) ≈ 2 m/s and its acceleration (∂*u*/∂*t*)|_τ = 0_ ≈ 300 m/s^2^. From these data we can estimate the time t*≈ 6.7 ms needed for the divergence to occur and the distance *z* ^*^≈ 13 mm from the orifice where the divergence first occurs. These parameters are close to the Rayleigh time and to the Rayleigh wavelength. This suggests that the jet becomes unstable simultaneously via a Rayleigh-Plateau instability and a kinematic gathering process. Quite interestingly each process, when considered alone, would produce a rather ineffective impact of the jet, due to the dispersion of energy in space and time associated with the kinematic gathering and the Rayleigh-Plateau instability, respectively. In fact, in the presence of a pure Rayleigh-Plateau instability the energy would be distributed in time within the stream of droplets generated by the instability, and the impact of such stream with the prey would be as ineffective as the impact of a cylindrical jet, due to the long impact time of the stream. This kind of behavior is apparent in the trailing part of the jet at the time of impact, which breaks down into a series of small satellite drops with a spacing compatible with λ_R_ (Fig. 1F). On the other hand, a pure kinematic gathering process would give rise to the spatial redistribution of the power of the jet over an ideally infinite surface perpendicular to the direction of propagation. Due to the finite size of the prey, the transfer of power would be limited to the portion of the front of the jet that actually hits the prey. In practice, the formation of a thin sheet of liquid with an infinite surface is prevented by the action of surface tension, which determines the accumulation of mass and momentum at the head of the jet in the form of a rounded bulge that gets progressively inflated by the gathering of sections coming from the trailing part of the jet. The combined action of kinematic gathering and Rayleigh-Plateau instability determines an accumulation of mass at the head of the jet, which gives rise to a dramatic decrease of the time Δ*t_i_* needed to transfer the momentum during the impact with respect to that associated with the impact of a cylindrical jet or of a stream of droplets. This decrease of the impact time Δ*t_i_* is the ultimate responsible of the hydrodynamic amplification mechanism described here.

### Conclusions

We have shown how the hydrodynamic amplification mechanism exhibited by the investigated specimens of *Toxotex jaculatrix* is the result of a subtle balance between surface tension and the inertia of the liquid. Such mechanism could represent a feature of the *T. jaculatrix* species, and possibly of other related species, such as *T. chatareus*. However, additional and more systematic studies on a larger set of individuals under well controlled breeding and growth conditions would be necessary to establish the general relevance of the mechanism. We note that an amplification mechanism such as that described here allows taking advantage of an expanded feeding niche. Quite interestingly, at variance with the catapult mechanism in chameleons, this advantage would come without the evolutionary cost for the development of highly specialized internal structures dedicated to the storing of mechanical energy. Many animals have the ability of throwing objects at a distance [30]. In the case of archer fish the genus name *Toxotes* (Greek word for archer) is originated by the ability of shooting precisely aimed darts of water at preys. In retrospect, this name could be far sighted and well deserved, In fact, our results suggest that archer fish employ an external mechanism for the amplification of muscular power, just like an archer does with his bow. This feature, when proven to be a general feature of the species, would make the archer fish a notable example of animal making use of a highly sophisticated tool such as a hydrodynamic lever to achieve a powerful impact with its preys.

## Materials and Methods

### Ethics Statement

The observation of animals was conducted in accordance with international and local regulations. Animals were housed and handled in agreement with the Directive 86/609/EEC of the European Council and the Italian Decree-Law D.L.116/1992. Two specimens of *Toxotes jaculatrix* imported by a commercial supplier were kept together inside a tank filled with brackish water (brackish water density 1.010 g/cm^3^, temperature 28°C) and fed once a day with live crickets and sticks of fish food. Water level was set at 25 cm above the bottom of the tank. Water quality parameters were monitored daily and kept stable at values compatible with the fish species. The tank was illuminated with a 12-hour dark/light cycle. While filming, the tank was illuminated with a 300 W halogen lamp. The study was approved by the Ethics Committee of the Università degli Studi di Milano (http://www.unimi.it/ateneo/20138.htm).

### Fish Specimens

The study was performed on two specimens of *Toxotex jaculatrix* with a standard length of 67 mm and 61 mm, respectively. Data presented in Figs. 1–3 are relative to the 67 mm specimen. Its size closely matched those of the ones used in previous studies of the amplification mechanism [9]–[10]. This allowed us to take advantage of the accurate anatomical reconstruction previously performed by Elshoud and Koomen [10]. For completeness of information we report in Figure S1 the experimental data for the force and for the specific power of the second specimen.

### Acquisition of Movie Sequences of the Jet

The focus of the present study is on the identification and investigation of the hydrodynamic amplification of power occurring during the propagation of the jet. Such hydrodynamic process is fully deterministic and the selected methods allow a reliable estimate of the order of magnitude of the quantities involved in the amplification process. A systematic investigation of the variability of the results on a finer scale, as generated for example by the utilization of a larger number of specimens, would be beyond the exploratory purpose of this work. The jet was filmed by using an IDT M3 high-speed digital camera recording at 1000 frame/s. A side view of the propagation of the jet was accomplished by using the method adopted by Timmermans [19]. A horizontal slit of width 12 mm was mounted on the top of the tank hosting the fish. The slit was parallel to the transparent side of the tank used to film the fish, and the camera was aligned with the optical axis of the objective perpendicular to this side. The prey was fixed at a vertical level above that of the slit. The position of the slit was such that the fish, in order to be able to see the prey with both eyes, was obliged to align with its sagittal plane parallel to one side of the tank. Dropping into the tank either a cricket or a stick of fish food rewarded each successful shooting. The camera was mounted onto a H shaped bridge that allowed to align the optical axis of the camera with the position of the mouth of the fish at the time of the shooting. This reduced to a minimum the distortions due to refraction at the interface between air and water. The distance from the snout of the fish to the prey ranged from 97 to 153 mm. For smaller distances, fish preferred to jump out of water to capture their prey. The upper distance limit was dictated by the compromise of attaining a well-resolved characterization of the contour of the jet, free of optical distortions, together with a shooting distance as large as possible.

### Image Processing

**Figure 4:**
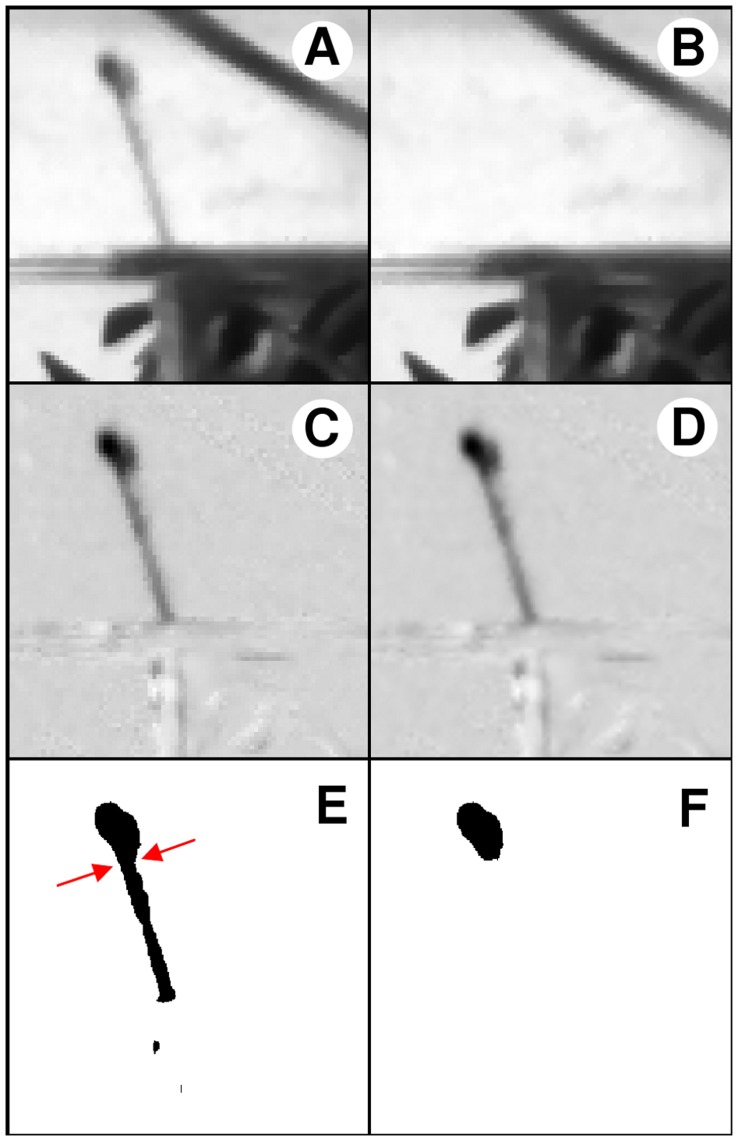
Image processing procedure. (A) original image. (B) background. (C) background subtraction. (D) oversampling 4×. (E) thresholding and binarization. Arrows mark the estimated position of the neckline. (F) projection of the head of the jet.

The analysis involved the processing of 30 image sequences showing a side view of the propagation of the jet. The image sequences were processed by using the FIJI image processing software (http://fiji.sc/wiki/index.php/Fiji), an enhanced distribution of NIH’s ImageJ, to determine the position and the outline of the head of the jet. The image resolution was 872×504 pixel and the size of a single pixel in real space was 0.5 mm. Images were digitized at 8 bit. The typical digitizing error due to camera noise was of the order of 3 bits. Sub-pixel resolution was achieved by oversampling the images by interpolating their intensity distribution. This allowed to achieve a sub-pixel resolution of the order of 10^−2^ mm. Distances were calibrated by using images of a reference pattern placed in the plane of the jet squirted by archer fish. We estimate that the evaluation of distances based on this calibration is accurate within 3%. Still features in the image sequences were removed by background subtraction (Fig. 4A–C). Typical results of the background subtraction procedure are also apparent in Fig. 1A–F, where one can identify only the moving features of the images, such as the fish, the prey and the jet.

Elevation angles of the jet over the horizon were determined by evaluating the angle comprised between the free surface of water and the line connecting the tip of the snout of the fish and the prey. Image sequences were spatially oversampled by a factor 4 (Fig. 4D) and then converted to binary images by using a thresholding procedure that produced a series of images of the black jet on a white background (Fig. 4E). The oversampling procedure allowed to limit the aliasing of the outline of the jet generated by the binarization procedure, so to obtain a well resolved contour line of the jet. The head of the jet was isolated from the rest of the jet by locating the pinch point connecting it to its tail (Fig. 4E–F). The transition point from the head to the tail was identified by visual inspection of each frame of the trajectory, because this procedure provided more reliable results than automated ones. Incidentally, a small error in the determination of the transition point does not affect to first order the estimate of force and power. This is due to the fact that both these quantities are determined by the ratio between the volume *V* of the head of the jet and its axial length *L*. As the volume is proportional to *L*, the ratio is not affected by small errors in *L*. This feature contributes to the robustness and reliability of the method employed to analyze the data. Image sequences of the movement of the head of the jet were processed automatically to determine the position of the centre of mass *X* of its projection, the volume *V* of the ellipsoid that best fitted the projected image, and the longitudinal extension *L* of the head of the jet in the direction of the motion. To recover the volume of the head from the two-dimensional projected image, the head was modelled as a prolate ellipsoid (Fig. 1I), under the assumption that the perturbation growing at the head of the jet exhibits radial symmetry due to the action of surface tension, because azimuthal perturbations would give rise to a larger surface area [20], [25]. We estimate that the uncertainty in the determination of the volume of the head of the jet is of the order of 10%. The data for *X*, *V* and *L* in the region where the head of the jet was not visible due to the presence of the slit were recovered by linear interpolation, followed by the addition of Gaussian noise. The width of the Gaussian noise was set to correspond to that of the experimental data in the region where the jet was visible. The moduli of the velocity *u* and acceleration *a* of the centre of mass were recovered by calculating the first and second time derivatives of *X*. A second order Savitzky-Golay filter was then applied to the data for *u*, *a*, and *V* to eliminate high frequency noise. The maximum average force *F* = ρ*Vu* ^2^/*L* that the head of the jet can exert onto a target and the mass-specific power *P*/*m*_m_ = 0.5 ρ*V u* ^3^/(*L m*_m_) were recovered from the smoothed data. Interpolation, smoothing, differentiation and filtering were performed by using National Instruments Labview 2010 (www.ni.com/labview) and OriginLab OriginPro 8 (www.originlab.com). In order to test and validate the image processing procedure we performed a systematic set of measurements by using a model system represented by a mechanically actuated piston that squirted a precisely controlled amount of water. The piston was actuated by a spring whose initial load was controlled accurately by a micrometer stage. The use of a transparent cylinder allowed us to determine the instantaneous outlet flow from the nozzle from the position of the piston. Both the piston and the outlet jet were filmed at 1200 fps by using a CASIO FX-1 camera to recover the time evolution of the position of the piston and of the head of the jet, together with the volume of the jet. This procedure allowed to perform a critical evaluation of the image processing procedure and to obtain a reliable estimate of the errors involved.

### Estimate of the Mass of the Muscles Involved in the Emission of the Jet

A detailed study performed by Elshoud and Koomen [10] investigated the action of the muscles involved in the squirting of the jet in archer fish. The use of electromyography allowed them to establish that 0.1 s prior to the emission of the jet the geniohyoideus muscle and the adductor operculi muscle are active to prevent outflow through the gill slit. The jet is then released by activation of the adductor arcus palatini and of the adductor mandibulae muscles, while the sternohyoideus muscle is not active before and during the emission of the jet. Elshoud and Koomen performed an accurate three-dimensional anatomic reconstruction of a 68-mm long specimen of *T. jaculatrix*. From their data, the total volume of the adductor mandibulae *A*_2_ *A*_3_, adductor arcus palatini, geniohyoideus, intermandibularis, dilator operculi and adductor operculi muscles involved in the emission of the jet is *V*_EK_ = 186.3 mm^3^. This volume must to be rescaled to take into account the different size of the specimens used in our study. The ratios *r* of the size of our specimens to that of Elshoud and Koomen are *r*_1_ = 0.985 and *r*_2_ = 0.987. Due to the small size ratio, we assumed for simplicity the isometric scaling of the volume *V*_VZC_ = *r* ^3^×*V*_EK_. By assuming a muscular mass density ρ_m_≈10^−3 ^g/mm^3^, the mass of the muscles delivering energy to the jet is *m*_m1_ = ρ_m_ *V*_VZC_ ≈178 mg for the first specimen and *m*_m2_ ≈135 mg for the second one.

### Impact of a Droplet onto a Solid Surface

The choice of the impact time as the time Δ*t* = *L*/*u* needed by the head of the jet to travel a distance *L* corresponding to its axial length implies that the molecules of liquid do not feel the presence of the target until they come into contact with it, thereby neglecting contributions due to the surface tension and to the viscosity of the liquid. This approximation is justified by recent work based on the pressure-impulse method [31], which shows that during the impact of a spherical drop the motion of the inertial flow perpendicular to the surface of the target stops in a time of the order of Δ*t* = *D*/*u* [23], [24], where *D* is the diameter of the drop. Therefore, although the typical time *L*/*u* slightly overestimates the impact time, it represents a conservative and reliable estimate of the impact time of the head of the jet in archer fish.

## Supporting Information

Video S1 Low-resolution color movie showing an archer fish shooting a jet of water to a prey supported by a clear plastic film. During the shooting the mouth of the fish barely emerges from the interface between water and air, while the rest of his body remains submerged. The image sequence has been grabbed at 400 frames/s and is played at 25 fps (QuickTime).
(MOV)

Figure S1 **Force and power at the head of the jet (specimen 2).** (A) time evolution of the maximum average force that the jet head can exert at the impact. The shaded area indicates the range of typical anchoring forces of insects such as flies, bugs and beetles. (B) time evolution of the mass-specific power that would be required by the muscles involved in the emission of the jet to accelerate the head in the absence of the hydrodynamic amplification process.
(TIF)
