# Supplementary figures and images for: How Archer Fish Achieve a Powerful Impact: Hydrodynamic Instability of a Pulsed Jet in Toxotes jaculatrix
Source: PLoS One. 2012 Oct 24;7(10):e47867. doi: 10.1371/journal.pone.0047867 (PMC3480456; doi:10.1371/journal.pone.0047867)

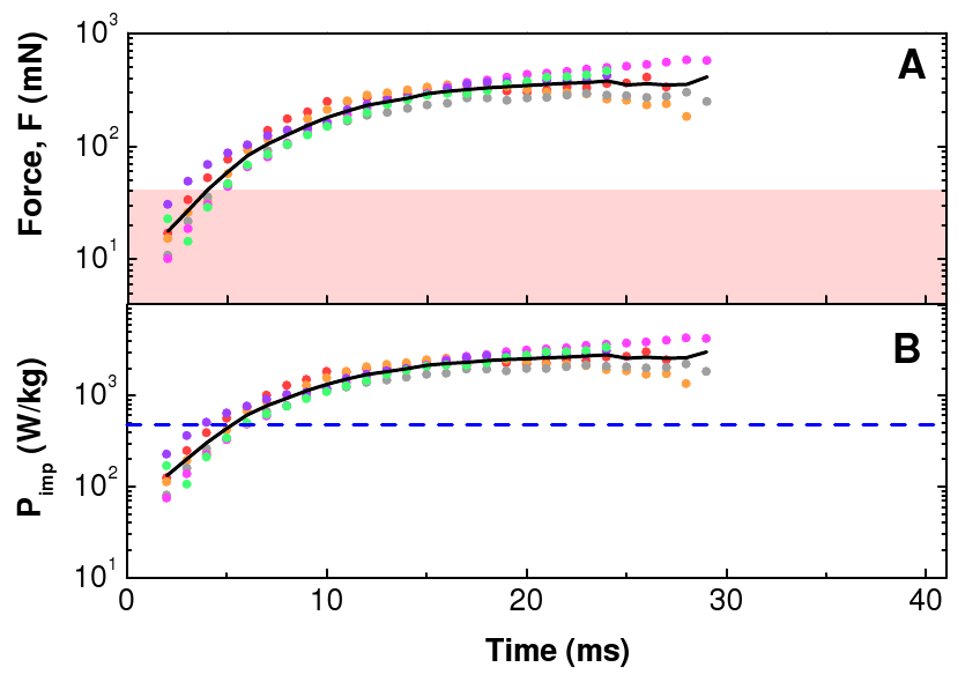

Supplement: Figure S1 — Force and power at the head of the jet (specimen 2). (A) time evolution of the maximum average force that the jet head can exert at the impact. The shaded area indicates the range of typical anchoring forces of insects such as flies, bugs and beetles. (B) time evolution of the mass-specific power that would be required by the muscles involved in the emission of the jet to accelerate the head in the absence of the hydrodynamic amplification process. (TIF) [file pone.0047867.s002.tif]
